# Natural Variation in Physicochemical Profiles and Bacterial Communities Associated with *Aedes aegypti* Breeding Sites and Larvae on Guadeloupe and French Guiana

**DOI:** 10.1007/s00248-020-01544-3

**Published:** 2020-07-03

**Authors:** Lyza Hery, Amandine Guidez, Audrey-Anne Durand, Christelle Delannay, Josiann Normandeau-Guimond, Yann Reynaud, Jean Issaly, Daniella Goindin, Grégory Legrave, Joel Gustave, Stéphanie Raffestin, Sebastien Breurec, Philippe Constant, Isabelle Dusfour, Claude Guertin, Anubis Vega-Rúa

**Affiliations:** 1 Laboratory of Vector Control Research, Transmission Reservoir and Pathogens Diversity Unit, Institut Pasteur of Guadeloupe, Morne Jolivière, Guadeloupe France; 2 Vector Control and Adaptation Unit, Cayenne, Institut Pasteur of French Guiana, Vectopôle Amazonien Emile Abonnenc, Cayenne, French Guiana France; 3 INRS-Centre Armand-Frappier Santé Biotechnologie, Laval, Québec Canada; 4 Laboratory of Environment and Food Hygiene, Institut Pasteur of Guadeloupe, Morne Jolivière, Guadeloupe France; 5 Regional Health Agency of Guadeloupe, Gourbeyre, Guadeloupe France; 6 Laboratory of Environment and Hygiene, Institut Pasteur of French Guiana, Cayenne, French Guiana France; 7 Transmission, Reservoir and Diversity of Pathogens Unit, Institut Pasteur of Guadeloupe, Pointe-à-Pitre, France; 8 Hyacinthe Bastaraud Faculty of Medicine, University of Antilles, Pointe-à-Pitre, France; 9 INSERM Centre for Clinical Investigation 1424, Pointe-à-Pitre, Les Abymes France

**Keywords:** *Aedes aegypti*, Breeding site, Physicochemical parameter, Bacterial community

## Abstract

*Aedes aegypti* develop in aquatic habitats in which mosquito larvae are exposed to physicochemical elements and microorganisms that may influence their life cycle and their ability to transmit arboviruses. Little is known about the natural bacterial communities associated with *A. aegypti* or their relation to the biotic and abiotic characteristics of their aquatic habitats. We characterized the physicochemical properties and bacterial microbiota of *A. aegypti* breeding sites and larvae on Guadeloupe and in French Guiana. In addition, we explored whether geographic location, the type of breeding site and physicochemical parameters influenced the microbiota associated with this mosquito species. We used large-scale 16S rRNA gene sequencing of 160 breeding sites and 147 pools of *A. aegypti* larvae and recorded 12 physicochemical parameters at the sampled breeding sites. Ordination plots and multiple linear regression were used to assess the influence of environmental factors on the bacterial microbiota of water and larvae. We found territory-specific differences in physicochemical properties (dissolved oxygen, conductivity) and the composition of bacterial communities in *A. aegypti* breeding sites that influenced the relative abundance of several bacteria genera (e.g., *Methylobacterium*, *Roseoccocus*) on the corresponding larvae. A significant fraction of the bacterial communities identified on larvae, dominated by *Herbiconiux* and *Microvirga* genera, were consistently enriched in mosquitoes regardless the location. In conclusion, territory-specific differences observed in the biotic and abiotic properties of *A. aegypti* breeding sites raise concern about the impact of these changes on pathogen transmission by different *A. aegypti* populations.

**Electronic supplementary material:**

The online version of this article (10.1007/s00248-020-01544-3) contains supplementary material, which is available to authorized users.

## Background

*Aedes aegypti* (Diptera: Culicidae) is a mosquito vector of arboviruses such as dengue, chikungunya and Zika that constitute major global health problems and threaten the French overseas territories of Guadeloupe and French Guiana [1, 2]. Besides the high susceptibility for these pathogens, this mosquito is a main vector of arboviruses because it is highly anthropophilic and *thrives* close to *humans* in urban and peri-urban areas [3, 4]. The species breeds mainly in human-made containers with volumes ranging from a few millilitres to hectolitres, such as drums, plastic buckets, cisterns, flower vases and rubber tyres [4, 5].

Water quality is critical for the choice of oviposition site for gravid females to ensure egg hatching and the development of their progeny from larvae to adults [6–8]. Females select breeding sites according to biotic and abiotic elements in the water, such as organic matter [8], bacteria [9, 10], phosphate, ammonia and potassium [11–13], which are known to be closely related to the abundance of larvae and adults in the field [13–15]. Larvae in their aquatic habitats rely on bacteria communities and organic matter whose composition is highly variable depending on environmental fluctuations [16, 17]. It has been well documented that environmental conditions such as nutritional deficiency, competition and high temperatures (> 30 °C) experienced during larval development can result in lifespan decrease, reduced adult size and increased susceptibility to virus transmission (i.e. Sindbis virus) [18]. In addition, exposure to bacteria in breeding sites during larval development shapes the microbiota of larvae and adult mosquitoes and also affects phenotype traits related to vectorial capacity, such as egg development, lifespan and vector competence [19–21]. For example, larval exposure to an *Enterobacteriaceae* isolate has been shown to reduce dengue virus dissemination titers in adult mosquitoes [16]. Similarly, exposure to a *Bacillus* isolate during larval development resulted in significantly increased rates of dengue virus infection and dissemination in the corresponding adults [22]. Thus, the quality of water in which *A. aegypti* mosquitoes breed might also have a role in determining their susceptibility to human pathogens. These findings highlight the importance of understanding natural variations in the habitat and microbiota of local *A. aegypti* populations and their potential contributions to adult phenotypic traits of epidemiological interest. However, the complexity and plasticity of *A. aegypti*–bacteria interactions in breeding sites are still poorly understood, and it is unclear how the bacterial communities in *A. aegypti* breeding sites are structured in natural settings at different geographical scales.

A handful of studies have reported marked effects of water physicochemical parameters (i.e. pH, dissolved oxygen) on egg hatch and larval physiology [23, 24], but information about their impact on vector competence is still lacking. Field and laboratory studies have shown that dissolved oxygen is positively correlated with larval abundance, while extreme salinity, temperature and pH reduce the abundance and development of larvae [25–30]. Interestingly, a few studies have shown that heavy metals such as iron (Fe), zinc (Zn) and copper (Cu) may be present at different concentrations in *A. aegypti* breeding sites [31, 32], but their association with the presence and abundance of *A. aegypti* larvae remains unclear [32]. Abiotic factors can also influence the structure of microbial communities in diverse ecosystems as soil and water. Pollution by heavy metals and changes in salinity [33], temperature [34] and pH [35] can change the metabolic activity and the relative abundance of bacteria. Changes in the microbial composition of the aquatic habitat and gut of mosquitoes have often been attributed to variations in geography [36, 37] or seasonal climatic patterns [38, 39], but no study has addressed whether changes in microbial composition are directly attributable to the physicochemical properties of breeding sites. Despite the omnipresence of *A. aegypti* mosquitoes in tropical and subtropical regions and their importance in public health, basic understanding of the abiotic factors and the natural microbial communities associated with local populations of the species and the corresponding aquatic habitats is still lacking.

We comprehensively characterized the physicochemical properties and natural bacterial communities associated with *A. aegypti* breeding sites and larvae on Guadeloupe and in French Guiana. We explored whether features of larval habitats, including geographical location, type of breeding site and the physicochemical parameters of the water (i.e. pH, temperature and turbidity) influence the variation in the bacterial microbiota associated with this mosquito species.

## Methods

### Collection of Samples

**Fig. 1:**
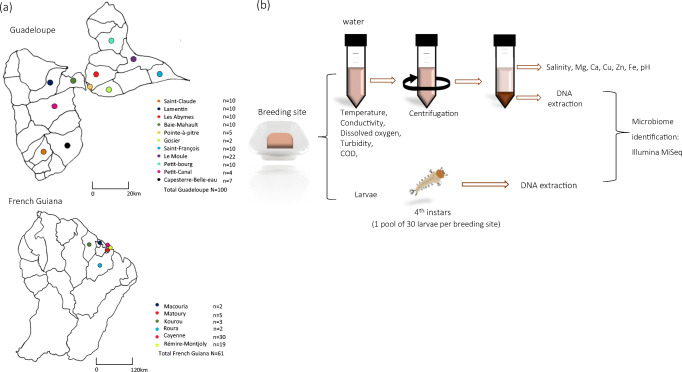
Study design. **a** Location of *Aedes aegypti* breeding sites sampled on Guadeloupe and in French Guiana. **b** Workflow for determination of breeding sites physicochemical parameters and for microbiota identification. N number of samples collected in municipalities

In 2017, *A. aegypti* breeding sites were selected in urban areas of Guadeloupe and French Guiana (Fig. 1a). At each breeding site (100 on Guadeloupe and 61 in French Guiana), sampling was conducted during the dry season: May and June on Guadeloupe and October and November in French Guiana. Artificial breeding sites (tyres, flower vases, drums, freezers) containing at least 100 *A. aegypti* larvae and a water volume of at least 700 mL were sampled. *A. aegypti* larvae were firstly identified in the field based on their specific movements and siphon morphology. The identification was then confirmed on the laboratory using a binocular loupe at a magnification of × 56 (Leica M80, Leica, Nanterre, France) and morphological descriptions [40].

The water and larvae were collected separately and taken to the corresponding facilities of Instituts Pasteur of Guadeloupe and French Guiana. The breeding sites were assigned to one of 10 classes according to their typology, in the categories used by local vector control authorities on Guadeloupe and in French Guiana [41]: (i) plant containers (pots, dishes, vases, basins), (ii) buckets (including water cans), (iii) drums, (iv) tyres, (v) cisterns, (vi) small waste (tins), (vii) large waste (freezers, toilet bowls), (viii) boats, (ix) water troughs and (x) gutters.

### Physicochemical Analysis of Water at Breeding Sites

Physicochemical analysis was performed on the water collected from breeding sites, as shown in Fig. 1b. For each breeding site, the water temperature (°C) (T), pH and electrical conductivity (μS/cm) were measured directly in the field with a multi-parameter probe (Odeon, PONSEL, France, on Guadeloupe and HANNA Instruments, France, in French Guiana) and an electrical conductivity meter (Cond 3210 + probe TetraCon 325, WTW, Germany). Dissolved oxygen (DO) (mg/L), turbidity (formazin nephelometric unit, FNU) and chemical oxygen demand (COD) (mg/L) were measured in 200 mL of water with no previous sample treatment. The remaining volume of water (~ 500 mL) was centrifuged at 8000 rpm for 10 min at 4 °C. The pellets obtained were preserved at − 20 °C for DNA extraction, and the supernatants were used to determine salinity (g/L), and Fe, Mg, Ca, Cu, Zn all measured in mg/L. All nine physicochemical parameters determined in the laboratory were analysed by accredited standard methods (www.cofrac.fr) at the Laboratory of Environmental Hygiene at the Instituts Pasteur of Guadeloupe and French Guiana.

### Bacterial DNA Extraction from Water and Larval Samples

For each breeding site, total DNA extraction was performed on 30 randomly selected fourth-instar larvae of *A. aegypti*, which were pooled and stored at − 20 °C, and on the pellets obtained from water samples. Total genomic DNA was extracted from the pellets with the NucleoSpin® Soil kit (Macherey-Nagel, USA) according to the manufacturer’s instructions (Fig. 1b). Pooled larvae were surface-sterilized as described previously [42] before DNA extraction. Briefly, each larval pool was first rinsed three times in 2 mL of sterile water, then exposed to 70% ethanol for 10 min. Lastly, the sample was rinsed six times: five in 2 mL of sterile water and once in 2 mL of sterile 0.8% NaCl. DNA extraction and PCR were carried out on each last water rinse to ensure the absence of exogenous bacterial DNA. Subsequently, each larval sample was crushed in 80 μL of sterile phosphate-buffered saline with a bead beater (MM 40, RETSCH, France) at 30 Hz for 30 s. Then, 20 μL of proteinase K (50 μg/mL) and 700 μL of SL1 (buffer lysis) from a NucleoSpin® Soil kit (Macherey-Nagel, USA) were added to homogenates, which were then incubated overnight at 56 °C. Samples were centrifuged for 2 min at 11,000 rpm, and the next steps of the protocol were carried out according to the manufacturer’s instructions. Sterile DNA-free water was used at each extraction as a negative control to check for contamination.

To confirm the presence of bacterial DNA in each water and larva sample, 25-μL PCR reactions were performed to amplify the 16S rRNA gene with the universal primers 27F (5′ GAGTTTGATCNTGGCGGCTCATCAG 3′) and 1492R (5′ GTNTTACNGCGGCKGCTG 3′), as previously described [43], using DreamTaq DNA Polymerase (Thermo Scientific, USA) according to the manufacturer’s instructions. The presence of PCR amplification fragments was confirmed on 1.5% agarose gel electrophoresis stained with Gel Red (Biotium, USA) and visualized under ultraviolet light.

### Illumina MiSeq Paired-End Sequencing and Sequence Processing

The V6–V8 region of the 16S rRNA gene was sequenced with the primers B969F-CS1 5′- ACGCGHNRAACCTTACC-3′ and BA1406R-CS2 5′-ACGGGCRGTGWGTRCAA-3′ [44]. The sequencing technology used was Illumina MiSeq 250-bp paired-ends, conducted at the Quebec Genome Innovation Centre (McGill University, Montreal, Canada), which generated 36,933,412 raw sequences from water (*n* = 161) and larval samples (*n* = 145). Sequences were processed with the software USEARCH version 10.0.240 following the UPARSE pipeline [45]. The two paired-end reads with fewer than five mismatches were merged. The maximum allowed ratio between the number of mismatched base pairs and the overlap length was set to 0.3. Reads with low-quality scores were removed, with a maximum expected error value of 1.0. The remaining 12,296,888 high-quality reads were de-replicated and sorted by abundance, and all singletons and chimera were removed. Unique reads were then clustered into operational taxonomic units (OTUs) with the UPARSE OTU clustering method and a 97% identity threshold, with a minimum of two sequences considered to be an OTU. Taxonomic assignment was realized with the Ribosomal Database Project classifier version 16 to remove OTUs identified as chloroplasts. OTUs represented by < 0.005% of the total number of reads were removed. The OTU table was normalized to 20,000 sequences/sample. Sequencing led to adequate coverage of the bacterial communities (Additional file 1).

### Data Analysis

Rarefaction curves were generated with the software SHAMAN (SHiny Application for Metagenomic ANalysis, Paris, France, shaman.pasteur.fr) to assess the sufficiency of sequencing [46]. The diversity of OTUs within and between samples was compared with alpha and beta diversity indices, respectively. Alpha-diversity metrics (species richness, equitability, Chao1, Shannon, Simpson diversity indices) and beta-diversity metrics (Bray-Curtis distances matrix) were generated with USEARCH. To summarize and compare the composition of bacterial communities at the different *A. aegypti* breeding sites and on larvae, bar charts and pie charts were generated showing the distribution of bacterial genera with SHAMAN. The physicochemical parameters and alpha diversity among different breeding sites and localities were compared in Kruskal-Wallis and Mann-Whitney *U* tests. *P* values were adjusted with a Benjamini-Hochberg correction.

Principal component analysis was used to explain variation in physicochemical parameters according to container type. A permutational multivariate analysis of variance analysis was conducted with Bray-Curtis distance matrices and 999 random permutations to determine the relations between categorical variables associated with each breeding site and the microbial communities identified from the corresponding water and larvae samples. The categorical variables tested were sampling locality, container type and physicochemical variable. Weighted and unweighted UniFrac distances were used to evaluate diversity among groups with non-metric multidimensional scaling plots in the R program Phyloseq [47]. Volcano plots were generated in SHAMAN to identify significant differences in abundant bacterial taxa in water and larval samples, and significant differences were determined by the Wald test. Canonical correlation analysis was used to identify among the 12 physicochemical parameters, those having a greater influence on the 100 most abundant bacterial genera in water and larval samples. Multivariate regression analysis was used to detect significant correlations between the physicochemical parameters selected from the canonical correspondence analysis and abundant bacteria genera. The results were displayed on a heat map. All tests were conducted with the software XLSTAT-Ecology (XLSTAT 2019.1.2), and the level of statistical significance in all analyses was *P* ≤ 0.05, except for the results of the multivariate regression analysis, for which the cut-off for significance was *P* < 0.15 in order to eliminate variables that were less strongly associated with microbial community members.

## Results

### Differences in Physicochemical Parameters of *A. aegypti* Breeding Sites on Guadeloupe and in French Guiana

**Fig. 2:**
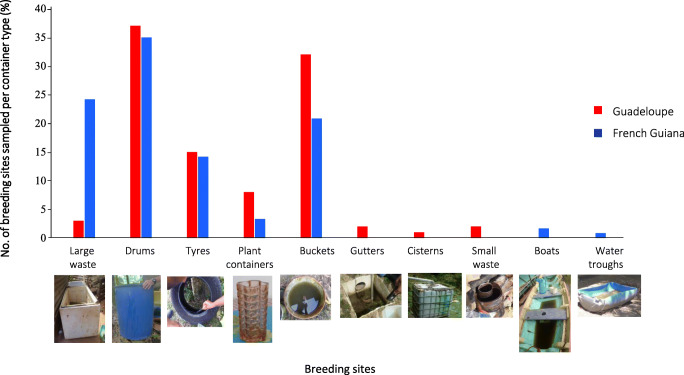
Breeding sites sampled per container type on Guadeloupe and in French Guiana

A total of 161 breeding sites in eight container classes on Guadeloupe and seven in French Guiana were investigated (Fig. 2). The most common breeding sites sampled were drums (> 30%), followed by buckets (> 25%) and large waste (~ 25% in French Guiana). Other breeding sites (tyres, plant containers, gutters, small waste, cisterns, boats and water troughs) represented < 15% in the two territories.

No significant differences were detected in pH or temperature according to container class or location (Table 1); however, an effect of container type was observed for electrical conductivity, turbidity, COD and mineral content, which were significantly higher in tyres, buckets and large waste than in drums. Differences were also observed between the two territories in levels of conductivity, dissolved oxygen, Ca and Mg in breeding sites, which were higher on Guadeloupe, and in turbidity, COD, Cu, Fe and Zn content, which were higher in French Guiana.

**Table 1:** Physicochemical parameters (mean ± SE) per container class on Guadeloupe (G) and in French Guiana (FG). Significant differences between G and FG for a given parameter and container type are shown in italic. Different letters indicate significant differences for a given parameter among container types in a same locality (G or FG) (*P* < 0.05). *COD*, chemical oxygen demand; *Cu*, copper; *Fe*, iron; *Zn*, zinc; *Ca*, calcium

Parameter	Locality	Large waste	Small waste	Buckets	Tyres	Drums	Water troughs	Plant containers	Boats	Gutters	Cisterns
Temperature (°C)	G	28 ± 1	29 ± 0.9	29 ± 1.6	30 ± 2	29 ± 1.8	_	29 ± 0.9	_	29 ± 0.5	26
	FG	28 ± 2	27	29 ± 2.5	28 ± 1.7	29 ± 2.1	24	_	34 ± 4.9	_	_
pH	G	8 ± 1	8 ± 1	8 ± 0.7	8 ± 0.5	8 ± 0.8	_	8 ± 0.5	_	7 ± 0.4	6
	FG	7 ± 0.5	7	7 ± 0.5	7 ± 0.7	7 ± 0.3	7	_	8 ± 0.9	_	_
Salinity (g/L)	G	0.2 ± 0.2	0	0 ± 0.1a	0.1 ± 0.1	0	_	0.1 ± 0.1	_	0	0
	FG	0.1 ± 0a	0.2abc	0abc	0.1 ± 0.1ab	0c	0.1abc	_	0.1abc	_	_
Conductivity (μS/cm)	G	464 ± 554abc	299 ± 205abc	*230 ± 215a*	304 ± 148ab	*96 ± 79c*	_	455 ± 230abc	_	202 ± 57abc	45abc
	FG	166 ± 179	356 ± 49	*105 ± 120*	213 ± 225	*35 ± 26*	236	_	199 ± 32.5	_	_
Dissolved oxygen (mg/L)	G	*10 ± 1*	9 ± 1	*9 ± 2.3*	*9 ± 1.4*	*9 ± 2.1*	_	9 ± 2.5	_	8.3 ± 1.8	9
	FG	*3 ± 1.7*	0	*4 ± 1.54*	*3.4 ± 1.8*	*4 ± 1.3*	9	_	6 ± 1.3	_	
Turbidity (FNU)	G	8 ± 5.7ab	47 ± 29.8ab	27 ± 28.2ab	*55 ± 29.9a*	16 ± 20b	_	46 ± 31ab	_	51 ± 30.7ab	7ab
	FG	49 ± 84a	133abc	18 ± 22abc	*120 ± 49b*	7 ± 8c	10abc		10 ± 6.7abc	_	_
Ca (mg/L)	G	17 ± 9.2abc	14 ± 7.8abc	*26 ± 36.2abc*	*35 ± 14.8a*	*11 ± 11.9b*	_	35 ± 19 ac	_	20 ± 19.1abc	2abc
	FG	11 ± 0.6a	18ab	*12 ± 17.5ab*	*11 ± 7.3ab*	*2 ± 1.9b*	_	_	20 ± 8.9ab	_	_
Mg (mg/L)	G	8.1 ± 10.5abcd	3.8 ± 2.2abcd	*4.7 ± 8.7a*	*3.5 ± 2.2ab*	*1.0 ± 01.3c*	_	7.0 ± 4.7abd	_	1.7 ± 0.3abcd	0abcd
	FG	1.4 ± 0.6	4.5	*1.5 ± 2.2*	*1.2 ± 1*	*0.2 ± 0.4*	2.3	_	2.3 ± 0.5	_	_
Cu (mg/L)	G	0.01 ± 0ab	0ab	0ab	0.02 ± 0a	0b	_	0.01 ± 0ab	_	0ab	0ab
	FG	0.03 ± 0abc	0abc	3 ± 9.9a	0.04 ± 0b	0 ac	_		0abc	_	_
Fe (mg/L)	G	*0ab*	2.7 ± 2.6ab	0.5 ± 1ab	*0.8 ± 0.9a*	0.3 ± 0.8b	_	0.3 ± 0.3ab	_	0.5 ± 0.3ab	0.1ab
	FG	*11 ± 26.6abc*	22.9abc	0.5 ± 0.4a	*10.9 ± 5.4b*	1.3 ± 2.4 ac	2.7abc	_	2.7 ± 0.9abc	_	_
Zn (mg/L)	G	*0ab*	0ab	0.3 ± 0.6ab	1.0 ± 1.3a	*0.5 ± 0.6ab*	_	0b	_	1.3 ± 1.7ab	3.1ab
	FG	*4 ± 6.7a*	0.2ab	0.5 ± 0.6b	2.7 ± 1.8ab	*1.3 ± 0.9ab*	_	_	1.3 ± 1ab	_	_
COD (mg/L)	G	21 ± 10.5abc	56 ± 46abc	46 ± 45a	195 ± 168b	*24 ± 30ac*	_	49 ± 25.6abc	_	20 ± 5.1abc	20abc
	FG	229 ± 426a	529abc	54 ± 43abc	216 ± 153ab	*17 ± 35c*	242abc	_	242 ± 149.2abc	_	_

**Fig. 3:**
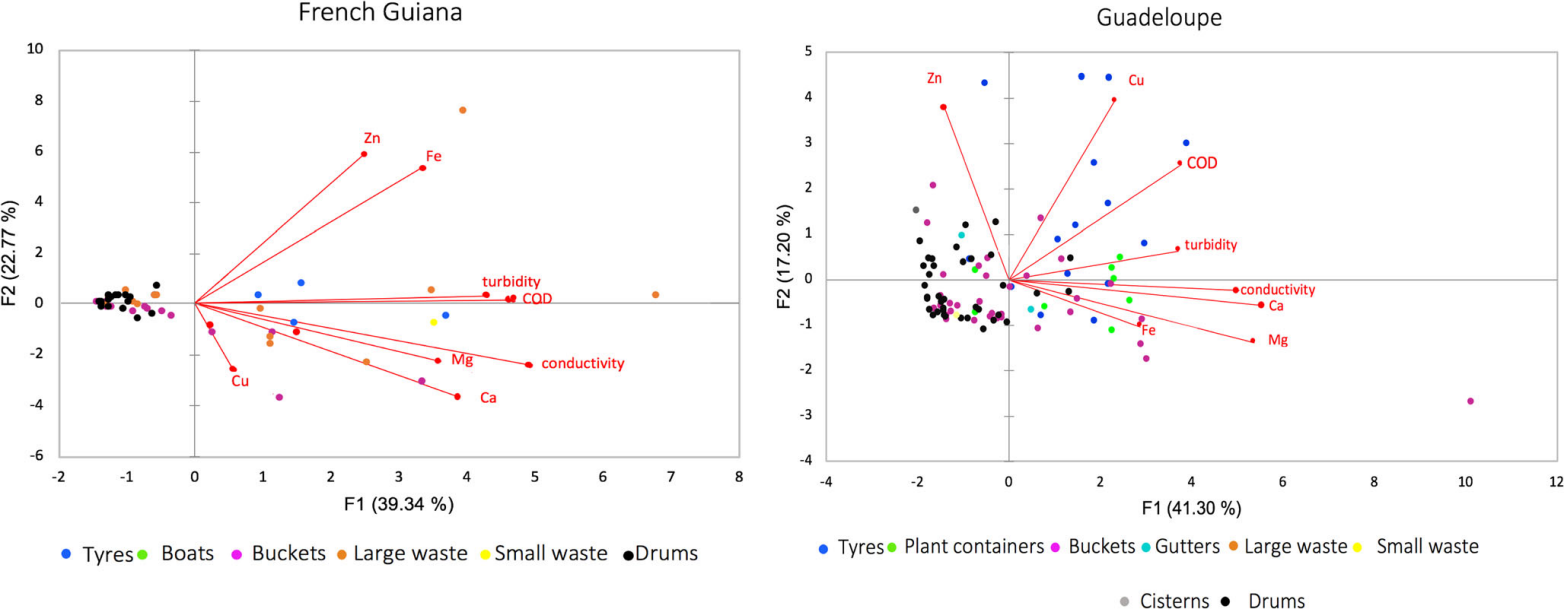
Principal component analysis of eight physicochemical parameters associated with *Aedes aegypti* breeding sites in French Guiana and on Guadeloupe. Dots correspond to the breeding sites sampled arrayed according to the measured physicochemical parameters. Colour-code indicates different container types. Vectors represent physicochemical parameters and point in the direction of steepest increase of values for the corresponding physicochemical parameter. The water samples from tyres and plant containers are closely related suggesting similar physicochemical profiles and located in the same direction as the vectors, indicating high COD, conductivity and turbidity. Conversely, the water samples from drums and buckets form a cluster on the opposite side of the vectors indicating lower physicochemical values. Ca, Calcium; Mg, Magnesium; Cu, Copper; Fe, Iron; Zn, Zinc; COD, Chemical oxygen demand. Percentages in parenthesis represent the variability explained by each principal component

The principal component multivariate analysis (PCA) revealed strong associations between physicochemical variables and breeding sites classes (Fig. 3), with 59% and 62% of the total variance explained by the first two axes on Guadeloupe and in French Guiana, respectively. Only eight physicochemical parameters out of the 12 measured displayed significant variation between sampling sites and/or container-types (Table 1), and were therefore selected for the PCA analysis. Whatever the sampling territory, the two main PCA components (F1 and F2) clearly separated breeding sites classes in two groups: one containing the tyres and the majority of the flowerpots (right area) and one containing the majority of buckets and drums (left area). Such structuration of samples suggests that physicochemical profiles in drums and buckets are similar, but differ from those of plant containers and tyres (PERMANOVA *R*^2^ = 0.22 for Guadeloupe, *R*^2^ = 0.23 for French Guiana, *P* < 0.001). Plant containers and tyres had indeed higher COD and conductivity when compared to other breeding sites classes, as reflected by the length and direction of the vectors for these two physicochemical parameters. Finally, the examination of physicochemical parameters associations revealed that across all types of containers, conductivity was positively correlated with Mg (Spearman *r* = 0.49, *P* < 0.01) and Ca (Spearman *r* = 0.62, *P* < 0.01), while turbidity was associated with COD (Spearman *r* = 0.59*, P* < 0.01). Zn content was positively associated with Fe in French Guiana (Spearman *r* = 0.64, *P* < 0.01) but negatively on Guadeloupe (Spearman *r* = − 0.23, *P* = 0.04).

### Bacterial Communities Associated with *A. aegypti* Breeding Sites

**Fig. 4:**
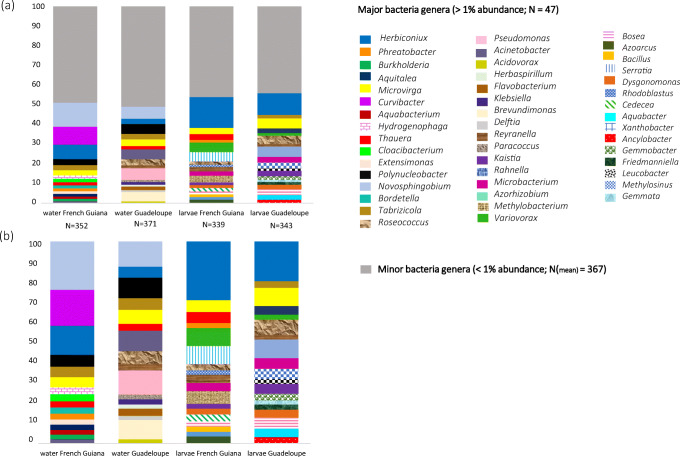
Mean relative abundance of bacterial genera found in *Aedes aegypti* breeding-sites water and larvae in Guadeloupe and French Guiana (**a**) and relative proportion of major bacteria genera (> 1%) (**b**)

**Fig. 5:**
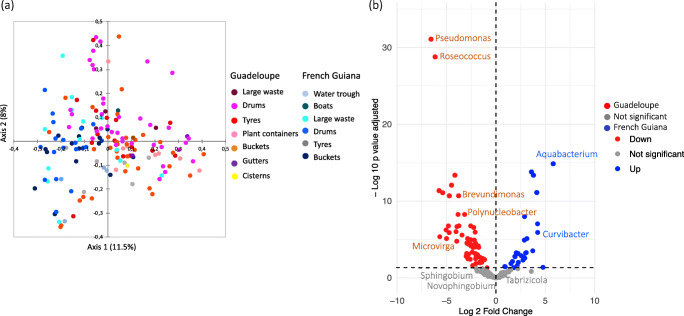
Spatial structuration and variability of microbiota associated with *Aedes aegypti* breeding site water. **a** Principal coordinate analysis plots based on Bray Curtis dissimilarity distances. Dots correspond to the breeding sites sampled arrayed according to the similarity of their microbiota. **b** Volcano plot for differential bacteria genera abundance in water from breeding sites on Guadeloupe and in French Guiana. Each dot represents a bacterial genera found in the samples and the most abundant bacterial genera, with a significant fold-change between the two territories (*P* < 0.05) are labelled

A total of 967 OTUs (117–540 OTUs per sample) belonging to 15 phyla and 376 genera were identified in water collected from *A. aegypti* breeding sites. The diversity and richness of the bacterial communities across breeding sites and localities were comparable (mean Shannon index 2.5 ± 0.9, mean richness 252.5 ± 0.16, Additional file 2), except in plant containers, which were significantly more diverse than the other classes of breeding site (ANOVA, Tukey post hoc*, P* < 0.05). The predominant phyla were *Proteobacteria* (54%), *Bacteroidetes* (22%), *Actinobacteria* (10%) and *Firmicutes* (6%) (Additional file 3). At higher taxonomic resolution, > 80% of the bacterial genera displayed low abundance in water (< 1%). The distribution of the 26 most abundant genera varied considerably among samples, with some genera (e.g., *Novosphingobium*, *Tabrizicola*, *Acinetobacter*) abundant in all containers and locations and others specific to the territory or habitat (Fig. 4, Additional file 4). Non-metric multidimensional scaling unweighted UniFrac analysis and PCoA plots based on Bray-Curtis dissimilarity revealed clear groupings of water samples by territory, showing significant differences in microbial composition between Guadeloupe and French Guiana (PERMANOVA, *R*^2^ = 0.2, *P* < 0.001, Fig. 5a, Additional file 5b). The differences were due mainly to taxa such as *Roseoccocus*, *Pseudomonas* and *Polynucleobacter*, which were significantly more abundant on Guadeloupe (log_2_ fold-change = 6.5, 5.5 and 5.21, respectively, *P* < 0.05), while *Curvibacter*, *Aquabacterium* and *Burkholderia* were more abundant in French Guiana (log_2_ fold-change = 3.3, 2.8 and 2.7 respectively, *P* < 0.05) (Fig. 5b, Additional file 6). On a finer scale, the bacterial communities in breeding sites were variable, and only slight structuring according to container type was detected (PERMANOVA, *R*^2^ = 0.14, *P* < 0.001, Fig. 5a).

### Bacterial Communities Associated with *A. aegypti* Larvae

Analysis of the microbiota of *A. aegypti* larvae revealed 931 OTUs belonging to 15 bacterial phyla and 346 genera. The sampled larvae harboured a wide range of bacterial taxa (up to 170 different species per sample), of which about 16% had a relative abundance > 1%. All bacteria taxa identified in *A. aegypti* larvae were found in the corresponding breeding sites. As for the water samples, the most abundant phylum in larvae was *Proteobacteria* (> 50% of all bacteria), followed by *Actinobacteria* (30%) and *Firmicutes* (10%) (Additional file 3). The bacterial communities of *A. aegypti* larvae were less rich (Kruskal-Wallis: *P* < 0.05) and more homogeneous than those in water from breeding sites, regardless the container type (Additional file 2). No major differences in larval microbiota were detected with respect to species composition, diversity (mean Shannon index 2.5 ± 0.9) or richness (mean 195.2 ± 0.35) in either territory (PERMANOVA, *R*^2^ = 0.03, *P* < 0.001, Additional file 2, Fig. 6a). The most widely spread, abundant bacterial genera were enriched in all the larval samples, regardless of locality, including *Bosea* (log_2_ fold-change over water = 3.3, *P* < 0.05), *Herbiconiux* (log_2_ fold-change over water = 3.2, *P* < 0.05), *Bacillus* (log_2_ fold-change over water = 3.5, *P* < 0.05) and *Kaistia* (log_2_ fold-change over water = 2.7, *P* < 0.05) (Table 2).

**Fig. 6:**
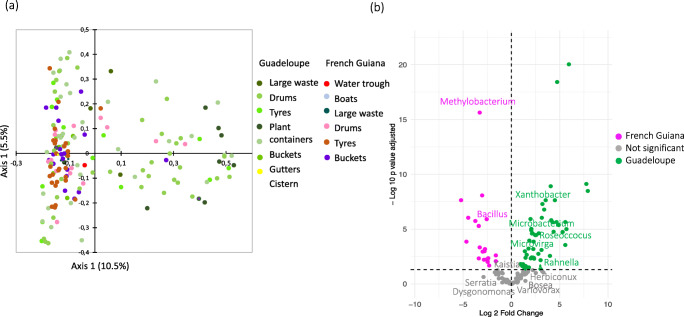
Spatial structuration and variability of microbiota associated with *Aedes aegypti* larvae. **a** Principal coordinate analysis plots based on Bray Curtis dissimilarity distances. Dots correspond to the breeding sites sampled arrayed according to the similarity of their microbiota. **b** Volcano plot for differential bacteria genera abundance in larvae between Guadeloupe and French Guiana. Each dot represents a bacterial genus found in the samples and the most abundant bacterial genera, with a significant fold-change between the two territories (*P* < 0.05) are labelled

**Table 2:** Bacteria genera enriched in *A. aegypti* larvae as compared to the corresponding breeding site

Similar enrichment in both territories (G vs FG, *P* < 0.05)	log_2_ fold-change	More enriched in G (G vs FG, *P* < 0.05)	log_2_ fold-change	More enriched in FG (G vs FG, *P* < 0.05)	log_2_ fold change
*Acidiphilium*	3.024	*Agromyces*	2.143	*Acidisphaera*	5.784
*Alsobacter*	3.359	Ancylobacter*	2.130	*Bdellovibrio*	5.106
*Bacillus*	3.545	*Aquisphaera*	2.062	*Legionella*	3.922
*Bosea*	3.333	*Bradyrhizobium*	4.942	Methylobacterium*	4.963
*Enterococcus*	2.459	*Caldilinea*	3.65	*Methylovirgula*	2.918
*Friedmanniella*	2.628	*Defluviicoccus*	3.473	*Nocardia*	3.392
*Gemmobacter*	3.275	*Desulfuromusa*	2.15	*Nocardioides*	4.011
*Herbiconiux*	3.252	Gemmata*	3.304	*Psychroglaciecola*	2.113
*Kaistia*	2.754	*Gemmiger*	3.417	*Romboutsia*	2.716
*Nitratireductor*	4.233	*Hyphomicrobium*	3.802	*Roseiarcus*	4.034
*Nitrolancea*	3.815	*Isoptericola*	2.462	Roseococcus*	5.93
*Rhodoblastus*	2.33	*Jiangella*	2.95	*Thauera*	3.302
*Roseomonas*	5.648	*Kinneretia*	1.929		
*Schlesneria*	2.545	*Leucobacter*	4.972		
		*Litorilinea*	3.115		
		*Methylocella*	2.989		
		Microbacterium*	2.109		
		*Mycobacterium*	2.062		
		*Pleomorphomonas*	2.109		
		*Rhodobacter*	4.105		
		*Saccharomonospora*	1.328		
		*Sphingobium*	3.207		
		Xanthobacter*	3.225		
		Xanthomonas	3.14		

(log_2_-fold change > 1; *P* < 0.05)

*Genera significantly more abundant in breeding sites in the corresponding territory *G*, Guadeloupe; *FG*, French Guiana

Territory-specific differences in relative abundance were detected for certain bacteria genera. For instance, *Methylobacterium* and *Bacillus* were significantly more abundant in larvae in French Guiana (log_2_ fold-change = 3.2 and 2 respectively, *P* < 0.05), while *Leucobacter*, *Friedmanniella* and *Xanthobacter* were more strongly associated with larvae on Guadeloupe (log_2_ fold-change = 5.7, 3.2 and 3.2, respectively, *P* < 0.05) (Fig. 6b, Table 2, Additional file 6). Interestingly, *Xanthobacter*, was also significantly more abundant in the water samples from Guadeloupe when compared to those from French Guiana (Additional file 6). Other genera, such as *Polynucleobacter* and *Emticicia*, predominated in water from breeding sites, while their relative abundance was significantly lower in the corresponding larvae (log_2_ fold-change = 9.196, and 7.945 respectively, *P* < 0.001) (Additional file 6).

### Relations Between the Composition of Bacterial Communities and Environmental Variables

We investigated whether differences in microbiota in water (*N* = 161) and larval (*N* = 145) samples were associated to by physicochemical variations. A single linear regression analysis identified the physicochemical parameters significantly associated with richness and diversity, and a multiple linear regression analysis of significant factors indicated the chemical parameters that better predicted microbiota structuration. On Guadeloupe, turbidity was significantly correlated with the bacterial diversity (*R*^2^ = 0.27, *P* < 0.05) and richness (*R*^2^ = 0.35, *P* < 0.05) in water samples, as well as with larval bacterial richness (*R*^2^ = 0.49, *P* < 0.05), while COD was slightly correlated with larval bacterial diversity (*R*^2^ = 0.10, *P* < 0.05). In French Guiana, turbidity appeared to be related to the evenness of the breeding site microbiota (*R*^2^ = 0.249, *P* < 0.05), whereas in the larval microbiota neither species richness nor species evenness were associated to physicochemical parameters.

Canonical correlation analysis was used to identify correlations among the 12 environmental variables and the 100 most abundant bacteria genera in water (Additional file 7a) and larvae (Additional file 7b) at breeding sites in each territory. As expected, physicochemical parameters were more correlated with bacteria genera from water than with those from larval samples. The CCA plot indicates strong positive correlation between conductivity, Ca, Mg, oxygen dissolved and several bacteria genera such as *Acinetobacter*, *Pseudomonas*, *Gemmobacter*, *Polynucleobacter* and *Ancylobacter* either in water and larvae, while these parameters were weakly or negatively associated to other genera commonly enriched in larvae such as *Bacillus* and *Herbiconiux*. Salinity and turbidity were the parameters that correlated the least with bacterial microbiota in water samples, while in larvae, the weakest correlations were obtained with Fe, Zn and salinity. For these reasons, these latter physicochemical parameters were excluded from further analysis.

**Fig. 7:**
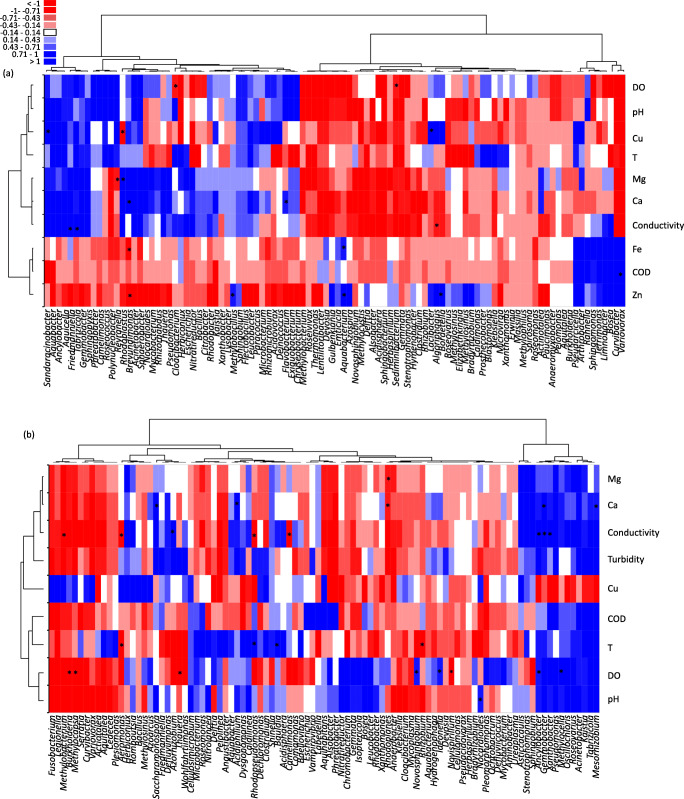
Heat map of correlations found by multivariate regression analysis between the 100 most abundant bacteria genera associated to *Aedes aegypti* breeding sites water (**a**) and larvae (**b**) (columns), and the physicochemical parameters retained after canonical correlation analysis (rows). Blue squares indicate a positive correlation in relative abundance, white squares indicate absence of correlation and red squares indicate negative correlations. The intensity of the colour corresponds to the magnitude of the (log)-fold change. Asterisks indicate significant correlations between a given bacteria genera and a physicochemical parameter (*P* < 0.05). COD chemical oxygen demand, DO dissolved oxygen, Cu copper, Fe iron, Zn zinc, Ca calcium

Multiple linear regression was then conducted using the retained potential microbiota determinants and the top 100 most abundant bacteria genera from water and larval samples (Fig. 7). The heatmap shows contrasted associations between DO, pH, Cu, Ca, Mg, conductivity, T and bacterial genera in water samples. Indeed, these parameters are positively correlated to *Sandaracinobacter*, *Aquabacter*, *Ancylobacter*, *Aquicella*, *Friedmanniella*, *Tabrizicola*, *Gemmobacter* and *Sphingopyxis* forming a clearly defined clade, while they are negatively correlated to a clade composed of 17 bacteria genera such as *Methylobacterium*, *Emticicia*, *Delftia* and *Aquabacterium* (Fig. 7)*.* Conversely, Fe, Zn and DCO are negatively correlated with bacteria in water samples, except for a clade where strong positive correlations are observed; these include *Curvibacter*, *Variovorax*, *Bosea*, *Limnobacter*, *Terrimonas*, *Sphingomonas*, *Rhanella*, *Arthrobacter* and *Pseudarcicella*. Regarding larval samples, two clearly defined clades being differentially influenced by physicochemical parameters are observed: (i) a clade composed by genera such as *Gemmobacter*, *Xanthobacter*, *Pseudomonas*, *Tabrizicola* and *Kaistia* that is positively influenced by the Mg, Ca, conductivity, turbidity, COD, T, DO, pH and (ii) a clade composed by *Fusobacterium*, *Serratia*, *Cedecea* and *Aquitalea* that is negatively impacted by these parameters. Interestingly, in these two clades, the associations obtained with Cu were contrasted with respect to those observed with the rest of physicochemical parameters. When considering the most statistically significant correlations (*P* < 0.05), conductivity and dissolved oxygen were associated to a higher number of bacteria genera in larval samples when compared to the rest of physicochemical parameters (Fig. 7).

## Discussion

The variability of *A. aegypti-*associated bacteria may trigger a differential influence on adult mosquito phenotypic traits such as survival and vector competence [16]. Hence, the characterization of habitat features influencing microbiota structuration is of major interest for risk assessments and vector control. In this study, we found that (i) the physicochemical properties and bacterial communities of *A. aegypti* breeding sites on Guadeloupe differ substantially from those in French Guiana, (ii) the container type strongly influences the physicochemical parameters but not the microbiota at breeding sites, (iii) the microbiota associated with *A. aegypti* larvae, even if influenced by that of the breeding sites, was highly conserved in both territories, and (iv) dissolved oxygen and conductivity are strongly associated with the composition of *A. aegypti* microbiota. The characterization was based on comprehensive sampling of 161 breeding sites conducted during the dry season in both territories to ensure that containers were not rinsed by rainwater and to better observe permanent breeding sites. We found that drums were among the most common *A. aegypti* breeding sites on Guadeloupe and in French Guiana, as was observed in the neighbouring regions of Martinique and Suriname [48–50]. Drums are used extensively to store water because of the irregular water supply, which provides an excellent habitat for *A. aegypti*.

An appropriate aquatic habitat for *A. aegypti* is regulated by biotic and abiotic factors and their interactions. As expected, the temperature and pH were generally around 29 °C and 7.5, respectively, regardless of the type of container and the territory, as they have been shown to be favourable for larval development and survival [51–53]. Dissolved oxygen, electrical conductivity, salinity, COD, turbidity and the concentrations of Ca, Mg, Fe, Zn and Cu depended on the territory and/or the container type, the highest values being associated with tyres and plant containers. The high content of mineral and organic compounds may be due either to the small volume of containers (i.e. tyres), the presence of plants or the container material (i.e. metal), which could result in a higher ion content, turbidity, COD and less dissolved oxygen [54–56]. Our results confirm that *A. aegypti* does not breed only in clear water, as it can breed in containers with high turbidity (> 133 FNU), high DCO (> 242 mg/L) and low dissolved oxygen, nearly reaching anoxia, like *Culex quinquefasciatus* mosquitoes [57]. These findings are consistent with reports of successful *A. aegypti* development in septic tanks and drums, indicating tolerance of high organic pollution [58], which, in the absence of a preferred habitat, allows *A. aegypti* to extend its niche and breed in marginal habitats to maintain its population, especially during unfavourable seasons. The differences found in the physicochemical parameters of breeding sites raise questions about their possible impact on the structure and composition of *A. aegypti* communities. Such interactions remain largely unexplored but should be considered, as habitat disturbance can alter the microbiota and thus affect adult vector phenotypic traits [59].

In agreement with other studies, we found that the predominant microbiota at *A. aegypti* breeding sites was Proteobacteria [17, 42, 60–67]. Significant differences in the relative abundance of bacteria such as *Pseudomonas* were recorded between Guadeloupe and French Guiana. Our regression analysis revealed positive correlations with Mg, Ca and conductivity*,* which is consistent with the higher abundance of these proteobacteria on Guadeloupe as they are known to be more abundant in environments with high electrical conductivity [68]. Of the proteobacteria, *Roseococcus*, *Pseudomonas* and *Brevundimonas* in particular are strict aerobic bacteria [68–70], which could explain the correlations obtained in our study between their abundance and higher dissolved oxygen, as seen on Guadeloupe. *Curvibacter* was particularly abundant in French Guiana breeding sites. These bacteria are frequently detected in iron-rich environments dominated by chemolithoautotrophic species or contaminated with toxic metals [71, 72]. In our study, *Curvibacter* were positively correlated with Fe and Zn, and negatively with dissolved oxygen, conditions that were characteristic of French Guiana, where large waste like abandoned, rusty freezers composed 24% of breeding sites sampled (only 3% of Guadeloupe breeding sites were large waste). Metal containers may have increased metal concentrations in the stagnant water contained therein as seen elsewhere [73]. Conversely, when considering the entire bacterial communities, the container type did not significantly influence the structure of the microbiota in breeding sites water or larvae.

By contrast to water samples, no major differences in species composition were observed in the microbiota associated with *A. aegypti* larvae between Guadeloupe and French Guiana (Fig. 5a). This is presumably due to host selection for microbial communities that can colonize the larval gut environment, which is consistent with the lower alpha diversity recorded in larvae when compared to water samples. The shared microbiota of *A. aegypti* larvae consisted of abundant genera such as *Herbiconiux*, *Bosea*, *Bacillus* and *Kaitsia*, which were more prevalent in the larvae than in the water from the corresponding breeding sites. *Herbiconiux* can degrade the cellulose and xylan found in the gut of some insects, which may explain the abundance of this ubiquitous genus in larval samples [74, 75]. The three other genera (*Bosea*, *Bacillus* and *Kaistia*) may be part of the core microbiota of mosquitoes, as they have also been identified in the gut of both larvae and adults specimens from other *Culex* and *Aedes* species [17, 62, 63, 76–78]. The role of these commonly found bacteria in gut vector biology may have led to their evolutionary conservation. While the functional roles of these genera are still largely unknown, *Bacillu*s are suspected of affecting the fertility of the mosquitoes [79], and their abundance and distribution were found to be associated to male mosquitoes and their particular feeding dynamics [80]. Interestingly, *Bacillus thuringiensis subsp. israelensis* (Bti) produce proteins with insecticide properties, which make it a widely used biological control agent [81].

Differences in the microbiota associated with *A. aegypti* larvae and those in breeding sites are also due to the relative abundance of bacteria which development is constrained by the larval gut environment. In our study, the aerobic bacteria *Polynucleobacter* and *Emticicia* bacteria were significantly more abundant in water samples from Guadeloupe than from French Guiana*,* but the relative abundance of these genera in the larvae were significantly lower than in the water they came from for both territories. As suggested by Coon and colleagues [82], the gut hypoxia, which is probably a cue for growth and moulting of larval mosquitoes, may constrain the growth of such aerobic bacteria. Nevertheless, how insects “select” and acquire from breeding sites the microorganisms that become part of their semi-stable microbiota is still not well understood.

Finally, we observed territory-specific differences in the relative abundance of certain bacteria genera in *A. aegypti* larvae (i.e. *Methylobacterium*, *Xanthobacter*, *Roseoccocus*, *Microbacterium*, *Microvirga*, and *Pseudomonas*) that were consistent with the differences between breeding sites on Guadeloupe and in French Guiana. These findings confirm that the larval microbiota are significantly affected by the microbial communities in water at breeding sites [16, 19]. As some bacteria from larvae can be transstadially transmitted to adult mosquitoes [83], the influence of breeding sites on mosquito microbiota raises questions about the transmission of pathogens. We found that *Pseudomonas* was more abundant at breeding sites and larvae on Guadeloupe than in French Guiana, whereas *Serratia* was more abundant in water and larvae from French Guiana. *Pseudomonas and Serratia* are genera commonly found both larvae and adult mosquitoes [42, 61, 64, 84–87], suggesting that the territory-specific differences observed on relative abundance of these genera at the breeding sites may be reflected in the microbiota of the corresponding adults and shape their transmission potential for pathogens.

Interestingly, contrasted abundances of *Serratia* and *Pseudomonas* genera were also found in adult *A. aegypti* from the Caribbean island of Grenada [88] but further studies using a standardized methodology would be required to assess their natural variability in mosquito populations across the Caribbean. Bacteria such as *Serratia marcescens* have been found to increase the susceptibility of *A. aegypti* females for dengue virus, while *Pseudomonas rhodesiae* can inhibit La Crosse virus replication in *Aedes albopictus* cells [89, 90]. *Bacillus* is another bacteria genus whose relative abundance was higher in French Guiana larvae than in those from Guadeloupe. This bacteria is also commonly found in adult mosquitoes [83] and has been found to decrease their susceptibility to *P. falciparum* infection [91]. It is noteworthy to mention that the vector competence experiments cited above used mosquitoes that were reared in laboratory conditions, and it is unknown whether the influence of *Serratia* spp., *Pseudomonas* spp. and *Bacillus* spp. on pathogen transmission by mosquitoes is maintained in natural conditions. Laboratory studies have also shown that *A. aegypti* from Guadeloupe and French Guiana have similar vector competence for arboviruses such as Zika and chikungunya [92, 93]; however, as larval exposure to bacteria can alter the vectorial capacity of this species [16], the differences we observed between Guadeloupe and French Guiana in terms of microbiota from breeding sites and larvae, may result in differences in the vectorial capacity of the corresponding *A. aegypti* populations.

## Conclusions

The physicochemical parameters at *A. aegypti* breeding sites depend on the type of container and the territory sampled; however, the only major differences on breeding site physicochemical profiles at a broader scale (between the two territories) were associated with differences in the relative abundance of genera in *A. aegypti* microbiota. How and to what extent geographical variation in microbiota at breeding sites and larvae affects the vectorial capacity of *A. aegypti* for human pathogens across the globe remain open questions.

## Electronic supplementary material

ESM 1 Rarefaction curves of OTU diversity for each sample. Each sample contains nearly 20, 000 sequences to ensure equal sampling depth. (PDF 1961 kb) (PDF 1960 kb)

ESM 2 Mean values for bacterial diversity and richness indices associated with water at breeding sites and *A. aegypti* larvae. (PDF 50 kb) (PDF 49 kb)

ESM 3 Relative abundance of the most abundant phyla. Bar shows mean relative abundance of the bacterial taxa sequenced from *A. aegypti* larvae and water samples from French Guiana and Guadeloupe. (PDF 1189 kb) (PDF 1188 kb)

ESM 4 Relative abundance of the most abundant genera. Bar shows mean relative abundance of the bacterial taxa sequenced from *A. aegypti* larvae and water samples from French Guiana and Guadeloupe. (PDF 1176 kbp) (PDF 1175 kb)

ESM 5 Spatial structuration and variability of microbiota associated with water at breeding sites and *A. aegypti* larvae. Non-metric multidimensional scaling plots based on UniFrac distances for water (A,B) and larvae (C,D) according to type of breeding site. (PDF 241 kb) (PDF 240 kb)

ESM 6 Significant differences on bacteria genera abundance according to sample type (water vs. larval samples) and/or the territory (XLS 34 kb). (PPTX 56 kb)

ESM 7 Canonical correlation analysis of 12 physicochemical variables and the 100 most abundant bacteria genera in *Ae. aegypti* breeding sites water (a) and larvae (b). COD, chemical oxygen demand; DO, dissolved oxygen; Cu: Copper; Fe: Iron; Zn: Zinc; Ca: Calcium. (PDF 33 kb) (XLSX 33 kb)
