# Supplementary figures and images for: Natural Variation in Physicochemical Profiles and Bacterial Communities Associated with Aedes aegypti Breeding Sites and Larvae on Guadeloupe and French Guiana
Source: Microb Ecol. 2020 Jul 3;81(1):93–109. doi: 10.1007/s00248-020-01544-3 (PMC7794107; doi:10.1007/s00248-020-01544-3)

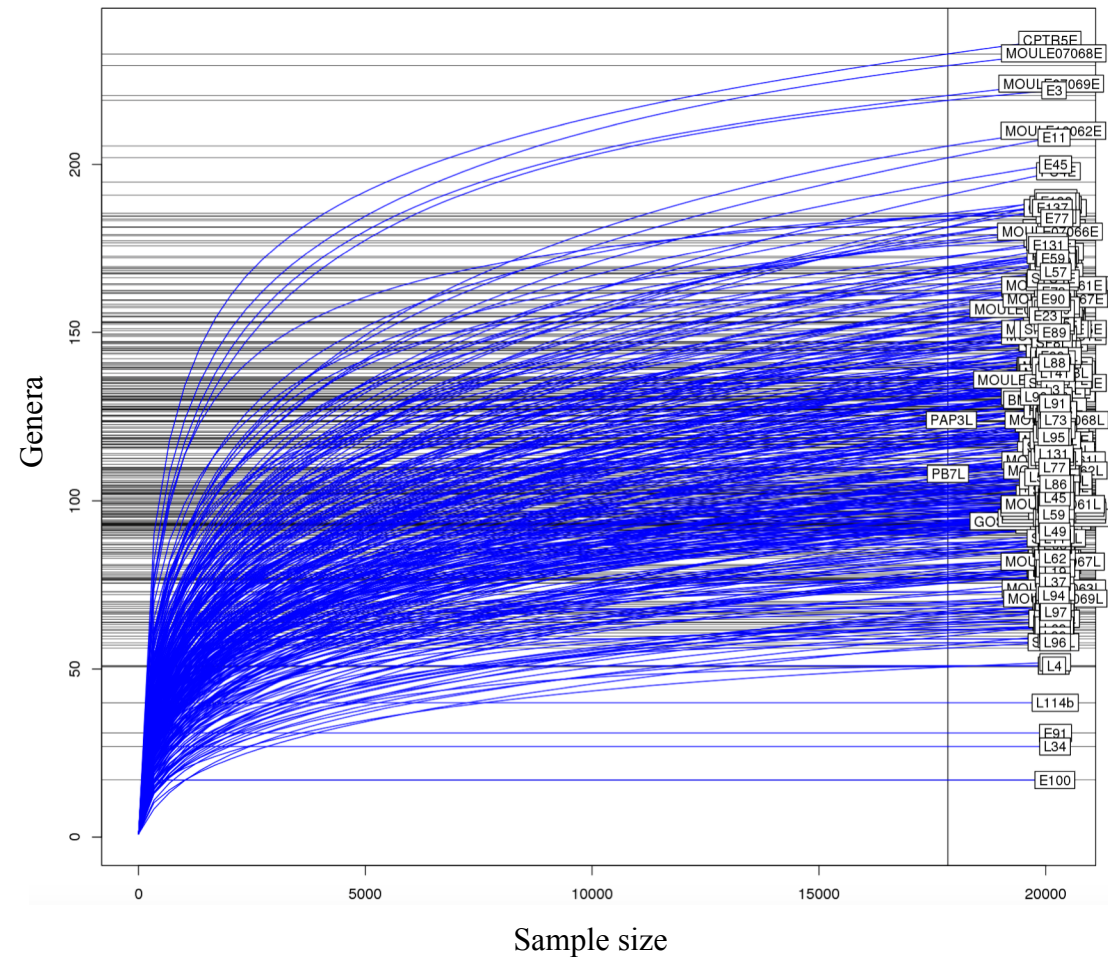

Supplement: Supplementary file 1 — Rarefaction curves of OTU diversity for each sample. Each sample contains nearly 20, 000 sequences to ensure equal sampling depth. (PDF 1961 kb) (PDF 1960 kb) [file 248_2020_1544_MOESM1_ESM.pdf]

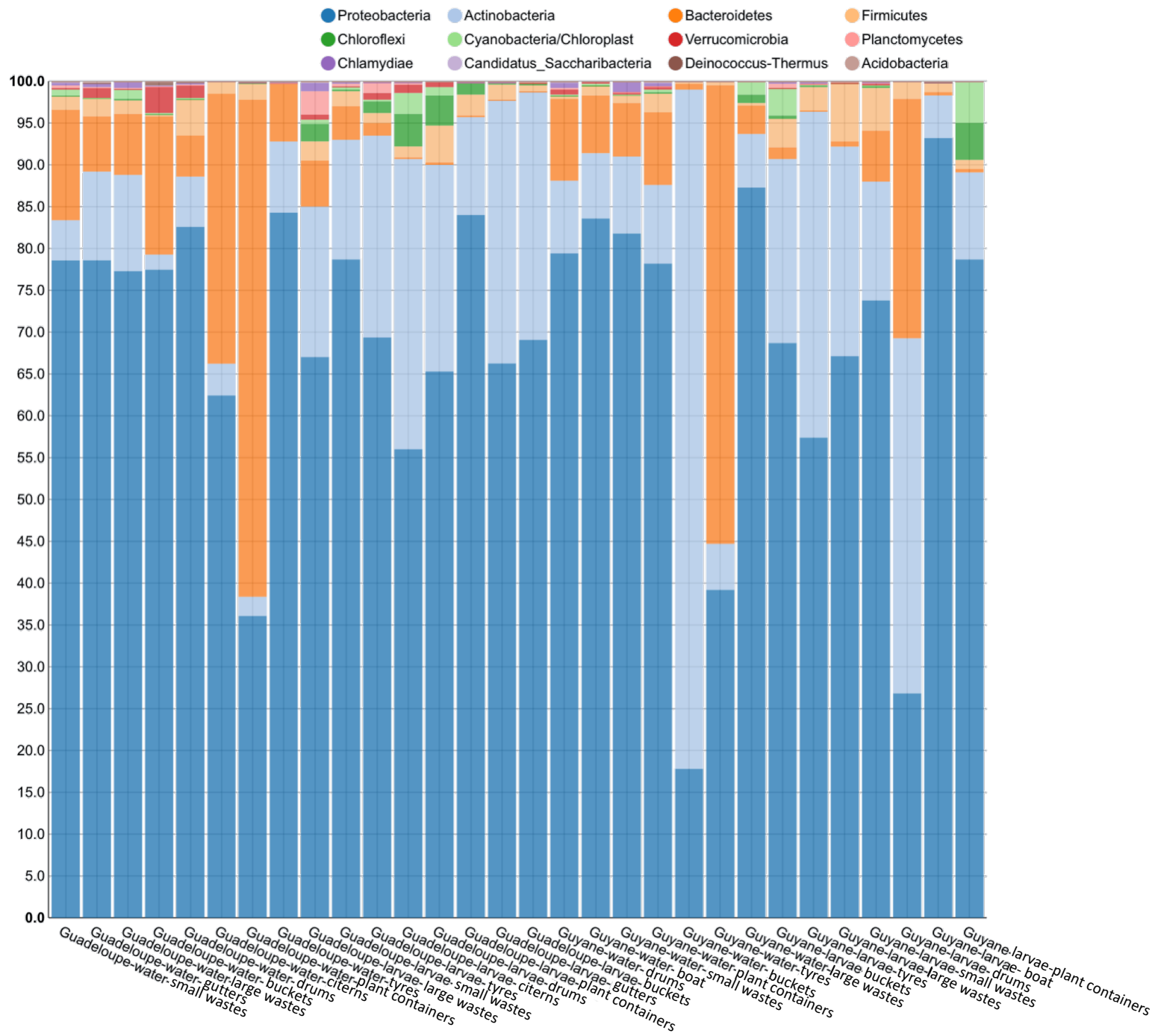

Supplement: Supplementary file 3 — Relative abundance of the most abundant phyla. Bar shows mean relative abundance of the bacterial taxa sequenced from A. aegypti larvae and water samples from French Guiana and Guadeloupe. (PDF 1189 kb) (PDF 1188 kb) [file 248_2020_1544_MOESM3_ESM.pdf]

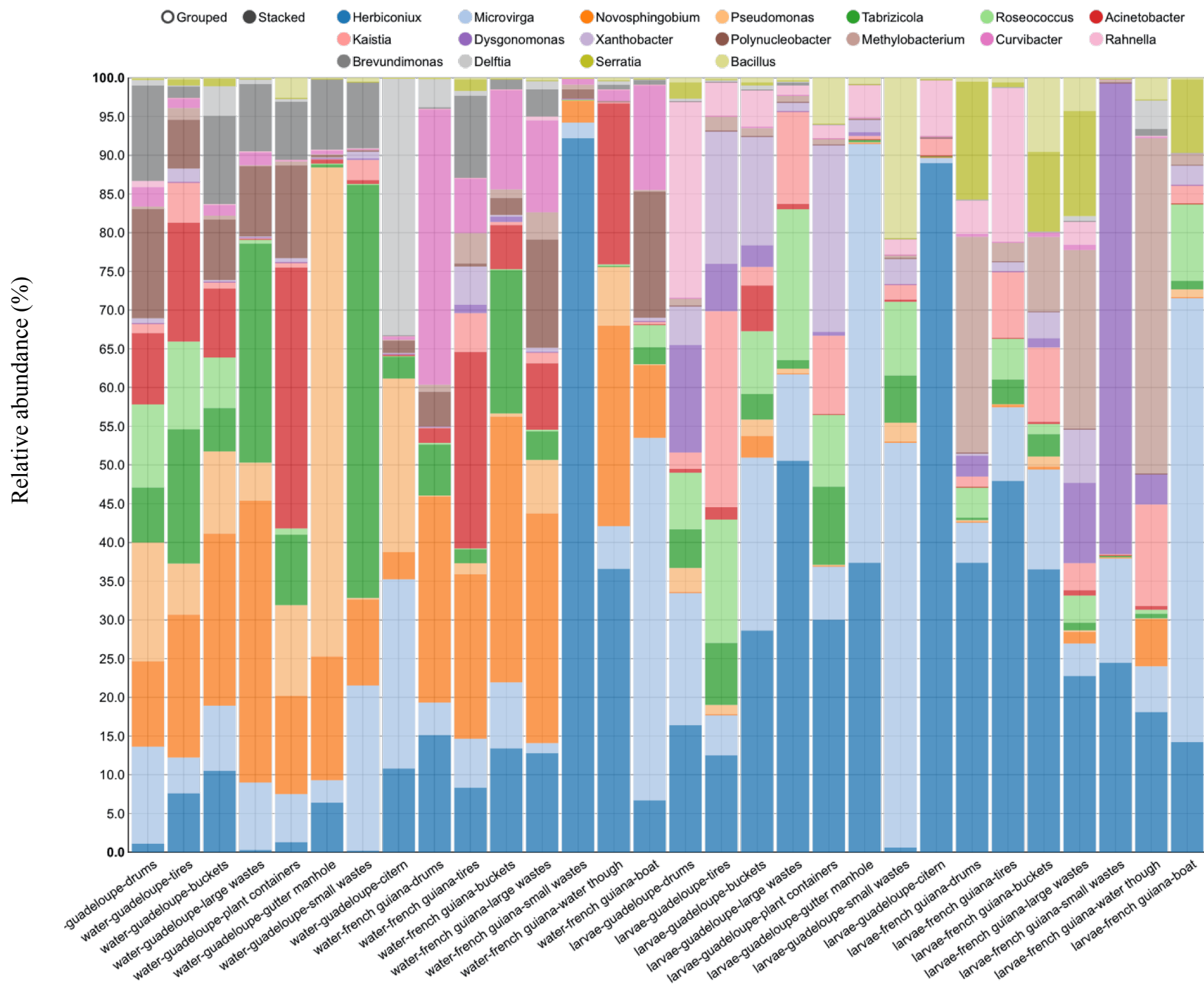

Supplement: Supplementary file 4 — Relative abundance of the most abundant genera. Bar shows mean relative abundance of the bacterial taxa sequenced from A. aegypti larvae and water samples from French Guiana and Guadeloupe. (PDF 1176 kbp) (PDF 1175 kb) [file 248_2020_1544_MOESM4_ESM.pdf]

## Slide 1
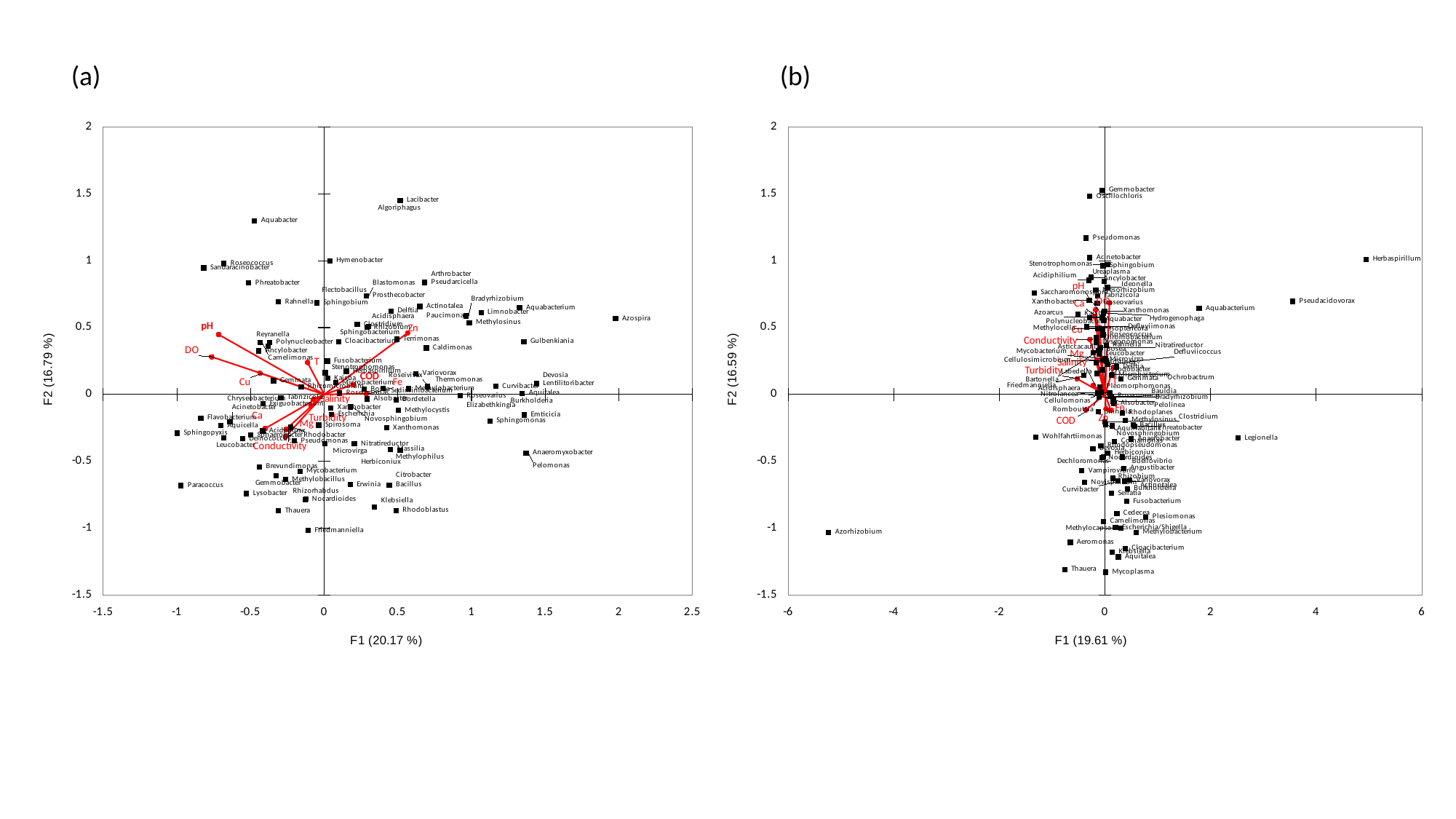

(a)
(b)
### Chart
| Category | | | | | | | | | | | | | | |
|---|---|---|---|---|---|---|---|---|---|---|---|---|---|---|
### Chart
| Category | | | | | | | | | | | | | | |
|---|---|---|---|---|---|---|---|---|---|---|---|---|---|---|

Supplement: Supplementary file 6 — Significant differences on bacteria genera abundance according to sample type (water vs. larval samples) and/or the territory (XLS 34 kb). (PPTX 56 kb) [file 248_2020_1544_MOESM6_ESM.pptx]
