# Supplementary material for: Natural Variation in Physicochemical Profiles and Bacterial Communities Associated with Aedes aegypti Breeding Sites and Larvae on Guadeloupe and French Guiana
Source: Microb Ecol. 2020 Jul 3;81(1):93–109. doi: 10.1007/s00248-020-01544-3 (PMC7794107; doi:10.1007/s00248-020-01544-3)
Supplement: Supplementary file 2 — Mean values for bacterial diversity and richness indices associated with water at breeding sites and A. aegypti larvae. (PDF 50 kb) (PDF 49 kb) [file 248_2020_1544_MOESM2_ESM.pdf]

Breeding site	Sample	Locality	Equitability	Simpson	Shannon	Chao1	Richness
Drums	Water	Guadeloupe	0.5	0.2	2.5	260.9	253.9
Gutters	Water	Guadeloupe	0.6	0.3	2.4	366.9	363.8
Cisterns	Water	Guadeloupe	0.5	0.1	3.5	280.2	274.3
Tyres	Water	Guadeloupe	0.5	0.2	2.9	263.5	259.3
Small waste	Water	Guadeloupe	0.4	0.3	2.3	249.7	248.0
Buckets	Water	Guadeloupe	0.5	0.2	2.6	258.4	254.6
Large waste	Water	Guadeloupe	0.5	0.2	2.7	240.2	236.5
Plant containers	Water	Guadeloupe	0.6	0.1	3.5	380.2	374.3
Drums	Water	French Guiana	0.5	0.2	2.6	210.1	207.8
Tyres	Water	French Guiana	0.7	0.1	3.7	285.2	283.8
Buckets	Water	French Guiana	0.5	0.2	2.7	240.2	236.5
Large waste	Water	French Guiana	0.5	0.2	2.5	260.9	253.9
Water trough	Water	French Guiana	0.5	0.2	2.9	263.5	259.3
Boat	Water	French Guiana	0.5	0.2	2.6	210.1	207.8
Large waste	Larvae	Guadeloupe	0.4	0.3	2.4	206.0	203.3
Drums	Larvae	Guadeloupe	0.5	0.2	2.4	185.4	180.9
Tyres	Larvae	Guadeloupe	0.5	0.2	2.5	213.3	204.7
Small waste	Larvae	Guadeloupe	0.5	0.2	2.5	223.3	220.1
Large waste	Larvae	French Guiana	0.5	0.2	2.5	195.8	199.5
Drums	Larvae	French Guiana	0.6	0.3	2.4	166.9	163.8
Tyres	Larvae	French Guiana	0.5	0.3	2.4	166.9	163.6
Buckets	Larvae	French Guiana	0.5	0.2	2.4	153.8	151.7
Water trough	Larvae	French Guiana	0.4	0.3	2.3	249.7	248.0
Boat	Larvae	French Guiana	0.5	0.2	2.6	167.0	167.0
