# Supplementary material for: Natural Variation in Physicochemical Profiles and Bacterial Communities Associated with Aedes aegypti Breeding Sites and Larvae on Guadeloupe and French Guiana
Source: Microb Ecol. 2020 Jul 3;81(1):93–109. doi: 10.1007/s00248-020-01544-3 (PMC7794107; doi:10.1007/s00248-020-01544-3)
Supplement: Supplementary file 5 — Spatial structuration and variability of microbiota associated with water at breeding sites and A. aegypti larvae. Non-metric multidimensional scaling plots based on UniFrac distances for water (A,B) and larvae (C,D) according to type of breeding site. (PDF 241 kb) (PDF 240 kb) [file 248_2020_1544_MOESM5_ESM.pdf]

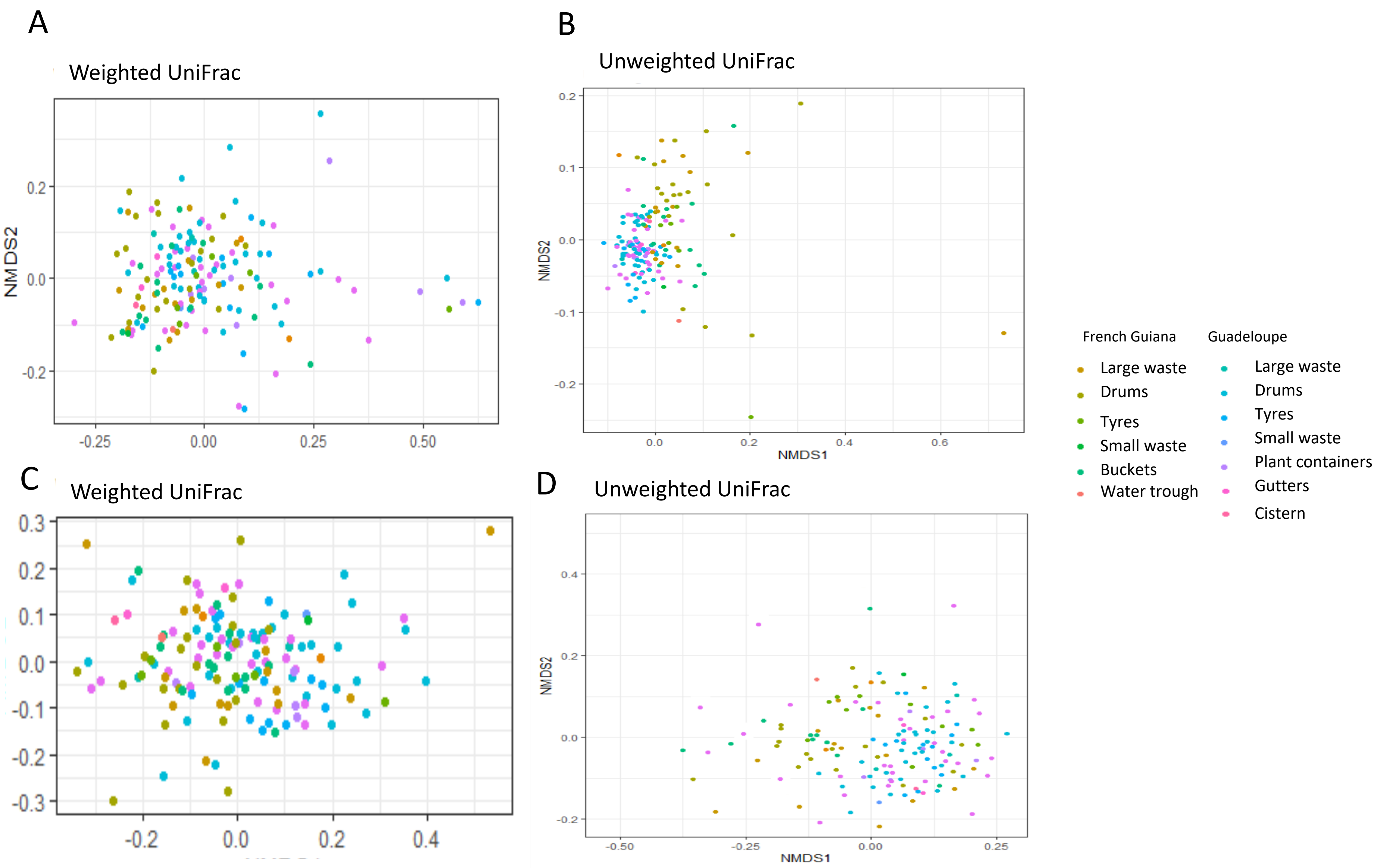

A
Weighted UniFrac
NMDS2
0.2
0.0
-0.2
-0.25
0.00
0.25
0.50
B
Unweighted UniFrac
NMDS2
0.2
0.1
0.0
-0.1
-0.2
0.0
0.2
0.4
0.6
NMDS1
C
Weighted UniFrac
0.3
0.2
0.1
0.0
-0.1
-0.2
-0.3
-0.2
0.0
0.2
0.4
D
Unweighted UniFrac
NMDS2
0.4
0.2
0.0
-0.2
-0.50
-0.25
0.00
0.25
NMDS1
French Guiana
Large waste
Drums
Tyres
Small waste
Buckets
Water trough
Guadeloupe
Large waste
Drums
Tyres
Small waste
Plant containers
Gutters
Cistern
